# Asymmetrical biomechanics in knee osteoarthritis: a review of contralateral risk from primary disease to post-arthroplasty progression

**DOI:** 10.1530/EOR-2025-0173

**Published:** 2026-06-01

**Authors:** Jing Dai, Jianxiong Ma, Bin Lu, Haohao Bai, Xinlong Ma

**Affiliations:** ^1^ Biomechanics Labs of Orthopaedics Institute, Tianjin Hospital, Tianjin University, Tianjin, China; ^2^ Tianjin Key Laboratory of Orthopaedic Biomechanics and Medical Engineering, Tianjin, China

**Keywords:** knee osteoarthritis, asymmetric biomechanics, total knee arthroplasty, symmetrical retraining

## Abstract

- Knee osteoarthritis (OA) demonstrates asymmetric joint degeneration (contrasting with symmetric inflammatory arthritis), driven by abnormal lower-limb biomechanics.
- Patients exhibit persistent inter-limb asymmetries in kinematics (reduced knee flexion/range of motion), kinetics (elevated knee adduction moment and increased contralateral loading), plantar pressure distribution, and muscle strength (15–30% quadriceps deficit), even with bilaterally matched disease severity.
- These asymmetries endure for months to years after total knee arthroplasty (TKA), manifesting as reduced weight-bearing on the operated limb and compensatory overloading of the contralateral limb.
- The high incidence of contralateral TKA (39% within 10 years) underscores the clinical importance of persistent biomechanical asymmetry as a prominent and modifiable contributor to OA progression in the opposite knee.
- Targeted rehabilitation strategies promoting inter-limb symmetry may mitigate disease advancement and improve functional outcomes.

## Introduction

Osteoarthritis (OA) is the most common joint disorder, characterized by degenerative changes in the articular cartilage. Knee OA involves not only cartilage degeneration but also pathological alterations in the synovium, menisci, musculature, and subchondral bone, all of which collectively contribute to the progressive deterioration of joint structure and function. Its primary clinical indications include joint discomfort, rigidity, and restricted joint mobility (1). OA commonly affects weight-bearing joints, such as the knees, hips, spine, and fingers. As per the global burden of disease study, knee OA constitutes roughly 85% of the overall burden of OA (2). Projections suggest that, by 2050, the global count of knee OA sufferers will reach an estimated 654 million (3).

Total knee arthroplasty (TKA) is a cornerstone treatment for advanced knee OA. The rising global prevalence of the condition is mirrored in the increasing demand for TKA. For instance, from 1990 to 2020, the age-standardized prevalence of knee OA rose by 56.9% (4). Accordingly, projections suggest a substantial rise in annual TKA volumes, with US estimates reaching about 1.22 million procedures by 2040 and nearly 2.92 million by 2060 (5).

Despite these trends and the well-established benefits of TKA for relieving pain and restoring function in the operated knee, a major postoperative concern remains. Evidence shows that the contralateral knee continues to be at high risk of disease progression (6, 7). In fact, OA in the contralateral knee often progresses more rapidly than in other lower-limb joints (8). Studies report that about 36% of patients may require a contralateral TKA within 10 years of their first surgery (9). This recurring pattern highlights a critical gap: we need a clearer understanding of the biomechanical factors that drive degeneration in the contralateral knee following unilateral TKA.

The knee joint, with its multi-compartment structure, is predisposed to medial compartment OA due to the significantly higher mechanical load transmitted through this region compared to the lateral compartment during normal activities (10, 11). Consequently, biomechanical factors are integral to the development of lower-limb OA, particularly in the pathogenesis of knee OA (12, 13). Abnormal biomechanical characteristics are frequently observed in OA-affected limbs, with an increased knee adduction moment (KAM) serving as a key indicator of elevated medial compartment loading (14, 15).

In contrast to systemic inflammatory arthritis, which typically presents with symmetrical joint involvement, knee OA is characterized by a predominance of asymmetrical joint pathology (8, 16). This asymmetry is closely linked to abnormal biomechanical patterns, manifesting as disparities in knee kinematics, kinetics, foot mechanics, and periarticular muscle function (17, 18, 19). Such biomechanical alterations have been implicated in the development of secondary OA in the contralateral knee (8, 20), as the unaffected limb often undergoes compensatory adaptations. Instrumented gait analyses comparing knee OA patients to healthy controls reveal that the contralateral limb demonstrates a significantly greater vertical ground reaction force (vGRF) and an altered plantar pressure distribution (21, 22). For instance, Worsley *et al.* documented that the contralateral limb in pre-arthroplasty patients bears higher vertical loads during walking than in matched healthy individuals, confirming absolute mechanical overloading (22).

Furthermore, altered movement patterns and muscle activation can impair dynamic joint stability – that is, the capacity to maintain control and proper alignment during activity. For example, patients who report knee instability show a reduced dynamic stiffness and abnormal flexion kinematics while walking, reflecting inadequate muscular stabilization (23). Deficits in quadriceps strength and proprioception have likewise been linked to poorer postural control on the contralateral side (24). Moreover, detailed kinematic studies detect abnormal tibiofemoral motion and disrupted neuromuscular coordination even during static single-leg stance (25).

Collectively, these alterations in joint mechanics and neuromuscular function show how biomechanical asymmetry creates an environment that predisposes the contralateral knee to degenerative changes, likely contributing to the progression of OA in a joint initially less affected. This chronic imbalance disrupts the knee’s mechanical homeostasis, worsening abnormal load distribution and increasing stress during movement (26). In advanced cases, this pathway can lead to the need for surgical intervention, including TKA, in the contralateral knee (8, 9, 27). Epidemiological evidence after TKA further supports this link: studies indicate that 39% of patients undergo contralateral TKA within 10 years, increasing to 45% within 20 years (28). These consistent findings highlight why addressing biomechanical asymmetry early in knee OA management is clinically important. Timely interventions to restore symmetry could, therefore, offer a promising way to lower the risk of contralateral joint degeneration and reduce the need for further surgery – ultimately supporting better long-term outcomes for patients.

**Figure 1:**
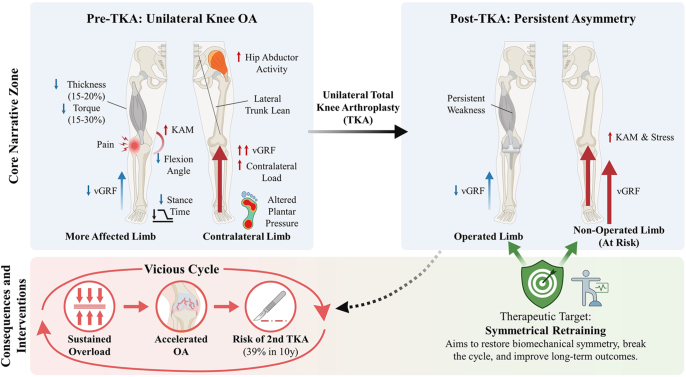
Pathomechanical cascade of asymmetry: drivers of contralateral knee risk from primary OA to post-TKA. (A) Primary knee OA: the more affected limb exhibits reduced knee motion, shorter stance time during walking, and weaker quadriceps strength. To compensate, the contralateral limb bears a higher vertical ground reaction force (vGRF) and knee adduction moment (KAM), leading to abnormal mechanical loading. (B) Following total knee arthroplasty (TKA): asymmetries often persist. The operated limb may remain weak (e.g. due to quadriceps impairment), leading to continued overloading of the non-operated side. This creates a self-reinforcing cycle that can accelerate OA progression in the contralateral knee. The schematic highlights symmetry-focused rehabilitation as a key therapeutic strategy to interrupt this cascade, restore inter-limb balance, and mitigate contralateral joint degeneration (created by the authors).

Therefore, to accurately assess this risk, the contralateral knee must be considered within two sequential clinical stages. In the first stage (asymmetric knee OA), the term refers to the less affected, typically nonsurgical knee in patients with unilateral or markedly asymmetrical disease. At this stage, abnormal biomechanics can initiate or accelerate degenerative changes in a joint that may be radiographically or clinically less involved. In the second stage (following unilateral TKA), the contralateral knee is the non-operated side. Here, persistent biomechanical asymmetries drive the progression of OA that is often already present. This review employs this two-stage framework to examine how biomechanical asymmetry initially elevates the risk for, and subsequently perpetuates the degeneration of, the contralateral knee throughout the disease continuum (Fig. 1). The schematic illustrates the pathomechanical cascade of asymmetry, highlighting key biomechanical parameters and therapeutic targets across both stages.

Patients with knee OA frequently exhibit biomechanical deviations across multiple joints during walking, which may serve as compensatory mechanisms to mitigate pain and reduce stress on the affected knee (29). However, these pain- and load-related adaptations alone do not fully explain the observed asymmetrical biomechanical patterns. Notably, even individuals with asymptomatic knee OA demonstrate significant asymmetry in lower extremity biomechanics, suggesting that factors beyond pain avoidance – such as structural joint changes, neuromuscular adaptations, or inherent gait variability – may contribute to these deviations (30). This highlights the complex interplay between symptomatic and biomechanical factors in shaping biomechanical asymmetries in knee OA, underscoring the need for a comprehensive understanding of their underlying mechanisms.

In summary, this review synthesized evidence from databases including MEDLINE, Embase, PubMed, and Web of Science, using search terms such as ‘gait’, ‘knee osteoarthritis’, ‘asymmetry’, ‘total knee arthroplasty’, and ‘biomechanics’. The search encompassed randomized controlled trials, meta-analyses, systematic reviews, and review articles. The findings are organized into two primary perspectives to elucidate the asymmetrical biomechanical manifestations of knee OA: (i) the asymmetric biomechanical manifestations of knee OA before treatment, including kinematic, kinetic, and muscle strength characteristics, and (ii) the asymmetric biomechanical characteristics of knee OA, particularly after TKA, with an emphasis on kinematic, kinetic, and muscle strength parameters. This review provides robust biomechanical evidence supporting the prevalence of inter-limb asymmetries in knee OA patients, underscoring the potential critical role of restoring symmetry in mitigating contralateral OA progression and improving gait rehabilitation outcomes. Future research should prioritize longitudinal studies to elucidate causal relationships and optimize targeted interventions for symmetry restoration.

## Asymmetric biomechanics in knee OA

### Joint kinematics and kinetics

Knee OA presents with characteristic asymmetries in both kinematics and kinetics. Patients typically exhibit a reduced knee flexion angle and shorter ground contact time on the affected side (29, 31). Extending beyond these localized changes, a reduction in hip internal rotation speed has also been observed in the more severely affected limb, indicating a broader pattern of kinematic asymmetry that involves the proximal joint (18). These alterations likely stem from compensatory strategies – such as adjustments in muscle activation, joint stiffness, and gait – that aim to reduce pain and off-load the compromised knee. Critically, such asymmetries are not confined to a single joint but reflect a dysfunction of the integrated lower-limb kinetic chain. This interconnectedness means that impairment in one region can alter loading and movement patterns throughout the limb, including on the contralateral side.

These biomechanical alterations are further evidenced by kinetic analyses revealing a significantly unequal force distribution between limbs (32, 33). The relationship between structural severity and biomechanical asymmetry is well established. As K&L (Kellgren–Lawrence) increases, asymmetric patterns become more pronounced during demanding activities, such as stair climbing (34). However, evidence regarding the manifestation of these asymmetries during walking relative to K&L grades remains limited, representing a critical knowledge gap.

The expression of these asymmetries, however, appears to be influenced by disease severity and laterality. Notably, in individuals with mild-to-moderate knee OA (K&L grades < 3), those with bilateral involvement demonstrate more pronounced between-limb kinematic asymmetries than their unilateral counterparts (18). In contrast, studies involving cohorts with more advanced OA (predominantly K&L grades 3–4) have reported that bilateral pain is associated with symmetrically altered biomechanics. In patients with unilateral pain, however, significant asymmetries are observed, including a greater varus angle and a lower external knee flexion moment in the painful knee (17). This pattern suggests that unilateral pain is associated with distinct biomechanical alterations, which may themselves be related to the underlying structural joint damage. Taken together, these contrasting patterns across disease stages may indicate a potential biomechanical evolution from asymmetry to compensatory symmetry as the disease progresses.

Multiple factors drive these asymmetries beyond simple pain avoidance. Severe OA patients adopt gait strategies that significantly reduce single-limb support time on the affected side (35, 36), while emerging evidence implicates neuromuscular adaptations, structural degeneration, and central sensorimotor reorganization in perpetuating asymmetric biomechanics (34, 37). The dissociation between pain relief and biomechanical normalization is particularly instructive: while Shin *et al.* observed reduced mechanical allodynia and weight-bearing asymmetry following pain signal inhibition (37), Ikeuchi *et al.* reported that intra-articular hyaluronic acid injections alleviated pain without resolving weight-bearing asymmetry (38). This suggests that compensatory mechanisms – including persistent neuromuscular guarding and contralateral limb overloading – may maintain asymmetries independently of nociceptive input.

Kinetic parameters provide particularly valuable insights into OA pathophysiology due to their direct relationship with intra-articular loading patterns. The asymmetric distribution of kinetic parameters, especially KAM, serves as a stronger predictor of OA severity and progression than purely kinematic measures (39). Elevated KAM asymmetries correlate with uneven medial compartment loading, accelerating cartilage wear in the more heavily loaded limb (40, 41).

The clinical significance of these kinetic asymmetries is substantial. Creaby *et al.* documented significant inter-limb asymmetries in both sagittal plane kinetics (reduced peak knee flexion moment) and frontal plane kinematics (elevated knee adduction angle), even in bilaterally involved patients (17). This mechanical imbalance results in a pathological contralateral overload. Worsley *et al.* demonstrated that the non-affected limb bore significantly higher loads during gait and sit-to-stand tasks. Critically, during sit-to-stand, loading on the contralateral limb exceeded not only the affected side but also healthy control values, indicating a clear pathological deviation. This overload state persisted for at least six months post-arthroplasty (22). Thewlis *et al.* corroborated this pattern, showing the contralateral limb supporting approximately 70% of total body weight during static stance in preoperative patients (42).

These asymmetries extend beyond ambulation to transitional movements. During sit-to-stand tasks, early-stage OA patients preferentially load the contralateral limb in mid-to-late movement phases, with the affected limb contributing only 30–40% of the total vertical force (43). This consistent pattern across functional activities underscores the systemic nature of load redistribution strategies in knee OA.

The persistence of biomechanical asymmetry in bilateral end-stage OA patients with matched pain severity (44) necessitates consideration of additional contributors beyond pain-mediated adaptations. Restricted joint mobility represents a key factor, as knee flexion contractures significantly alter force distribution. Harato *et al.* demonstrated that simulated unilateral knee flexion contractures reduce vertical forces on the restricted limb by 22–25% while increasing contralateral loading (45). Clinically, extension deficits exceeding 15° disrupt postural equilibrium, creating a 30–40% mechanical overload on the contralateral knee during walking (46, 47).

### Trunk movement

Knee OA induces asymmetrical biomechanical alterations that extend beyond the lower extremities to encompass compensatory trunk movement patterns. Iijima *et al.* demonstrated that patients with advanced knee OA (K&L grade 3) exhibit significantly greater trunk motion asymmetry in the medial–lateral plane compared to those with mild disease (K&L grade 1). Increased trunk motion asymmetry in severe knee OA (K&L grade 3) correlates with quadriceps strength disparities between limbs (48), suggesting that weakened quadriceps function on the affected side may drive compensatory postural adjustments (lateral trunk lean) to unload the degenerated joint. These adaptations, while initially protective, may perpetuate asymmetrical loading patterns across the lower extremities and trunk (48). In addition, the weakness of the hip abductor muscles, particularly the gluteus medius, could further contribute to lateral trunk lean (49). This compensatory strategy serves to maintain pelvic stability during single-limb support and is commonly observed in other gait pathologies, such as hip OA- and TKA-related limp (50). These findings are complemented by Turcot *et al.*’s observations of lateral trunk inclination toward the less affected limb during sit-to-stand transitions in OA patients compared to healthy controls (51), suggesting a systemic compensatory strategy to minimize mechanical stress on degenerated joints.

While these static and transitional movement analyses provide initial insights, the biomechanical implications of trunk asymmetry during ambulation remain poorly characterized. The current understanding of gait adaptations suggests that lateral trunk lean toward the stance limb may serve to reduce KAM by shifting the center of mass medially, potentially alleviating medial compartment loading (52, 53). Paradoxically, pain-avoidance behaviors in OA patients might conversely promote contralateral lean, inadvertently increasing load transfer to the unaffected limb (54). This unresolved dynamic – wherein trunk adaptations may simultaneously reduce KAM on the stance limb and increase load transfer to the contralateral limb – highlights a critical gap in the literature. Existing studies predominantly focus on discrete tasks (sit-to-stand) rather than cyclic locomotor activities, limiting our understanding of how dynamic trunk kinematics influence long-term joint loading asymmetry. Furthermore, the mechanistic interplay between neuromuscular compensation, structural degeneration, and habitual movement patterns remains underexplored.

### Foot biomechanics

During walking, the foot serves as the primary interface transmitting vGRF to the knee joint (55). Knee OA is associated with bilateral asymmetrical foot mechanics, which may reflect both compensatory adaptations to joint degeneration and potential drivers of disease progression. Chen *et al.* reported that patients with knee OA exhibit more severe foot posture asymmetry compared to healthy adults, characterized by a reduced stance phase duration and an uneven plantar pressure distribution on the affected limb (21). This asymmetry correlates with a poorer static stability and an increased fall risk, likely due to shifts in the body’s center of mass (21). Furthermore, foot posture asymmetry is positively associated with radiographic severity (K&L grade) in knee OA (56).

While foot asymmetry may arise as a secondary adaptation to knee pathology (pain avoidance or joint instability), it can reciprocally exacerbate mechanical imbalances. For instance, reduced ground contact time on the affected limb may increase contralateral limb loading during stance, elevating cumulative stress on the unaffected knee. This suggests a potential feedback loop – knee impairment (pain and instability) leads to adaptive gait changes and altered foot posture. These postural shifts, in turn, modify lower-limb kinetics and muscle function (56), which may redistribute joint loads and potentially exacerbate knee pathology over time (57). This bidirectional relationship complicates causal inference and highlights the need for longitudinal studies to elucidate the directional pathways within this foot–knee interaction. This proposed feedback loop remains a hypothesis to be tested by longitudinal studies integrating gait analysis and imaging. Additionally, dynamic biomechanical modeling could quantify how asymmetrical foot contact alters joint loading distribution across the lower extremities.

### Lower-limb muscle strength

In the biomechanics of knee OA, the strength of the joint muscles plays a crucial role, serving as one of the primary tissues for lower-limb movement. Muscles generate force through contraction and utilize the musculoskeletal system to execute various limb movements, maintaining body stability and balance. Therefore, studying the muscles around the joints of knee OA patients is crucial for a deeper understanding of the biomechanical mechanisms of knee OA (58, 59). The lower limbs of knee OA patients not only exhibit asymmetric kinematics and dynamics within the joints but also demonstrate asymmetry in muscle strength around the knee joints of both lower limbs (24, 60, 61).

Current research has consistently demonstrated significant inter-limb asymmetries in quadriceps function among patients with unilateral knee OA, with clinically meaningful deficits in muscle morphology, activation, and torque generation on the affected side. The affected limb exhibits a 15–20% reduction in quadriceps muscle thickness compared to the contralateral side, as measured by ultrasound imaging (24, 62). This morphological deficit is accompanied by a 20–25% increase in shear modulus, indicating a greater muscle stiffness and reduced compliance on the affected side (58). Maximal EMG activity of the quadriceps is 25–30% lower on the affected limb during voluntary contractions, reflecting impaired neuromuscular activation (54). Isometric torque production during maximal voluntary contraction (MVC) is 15–30% lower on the affected side, with deficits escalating during dynamic tasks (stair ascent) (63, 64). Furthermore, Rossi also found that the peak torque generated by knee extensors and flexors on the affected side was 76 and 82% of the unaffected side, respectively (60).

Muscle strength asymmetry in knee OA manifests across multiple lower-limb muscle groups, with distinct patterns in knee-focused and hip-focused musculature. Across OA severity groups (mild to severe), the affected limb gluteus medius strength is 12–18% lower, with asymmetry escalating alongside radiographic progression (K&L grade) (65). Such weakness may contribute to compensatory gait patterns, including a limp that is commonly accompanied by lateral trunk lean. In addition, the affected limb adductor strength is 9% lower than in the contralateral side, destabilizing pelvic control during single-leg stance (66). The interplay between knee and hip muscle asymmetries creates a self-reinforcing pathological cycle: reduced knee extensor torque forces reliance on hip abductors or adductors to stabilize the limb, accelerating hip muscle fatigue; gluteus medius weakness permits excessive femoral adduction, elevating medial knee joint reaction forces.

As in a prior study, knee OA patients have a reduced strength in muscles above the knee compared to the contralateral side, while muscles below the knee show different strength patterns. One study reported a higher gastrocnemius tension and Achilles tendon stiffness and resting tone on the more affected side in severe unilateral knee OA cases (56). It is speculated that the decline in thigh muscle strength (quadriceps) leads to a decreased knee stability, forcing the calf muscles (gastrocnemius–soleus complex) to enhance ankle stiffness via an increased tension and tendon stiffening to partly compensate for proximal functional deficits.

In knee OA, the functional use of the affected joint is significantly reduced due to pain, leading to disuse atrophy of the associated musculature on the affected side (67, 68, 69). This process of disuse atrophy on the affected limb may represent a key early contributor to the development of lower-limb asymmetry. During ambulation, patients exhibit an increased reliance on the unaffected limb, which further exacerbates the asymmetry in lower-limb muscle strength and function. Yoshida *et al.* have demonstrated that when the quadriceps of the affected limb are severely weakened, patients are compelled to adopt compensatory gait strategies, placing a greater mechanical load on the contralateral limb (70). Furthermore, lower-limb muscle asymmetry may be linked to alterations in central nervous system inhibitory mechanisms and changes in sensory input induced by chronic pain (71).

Knee OA patients frequently exhibit lower-limb asymmetry in muscle strength, biomechanics, and functional performance, which significantly influences disease progression and joint function. Unilateral quadriceps weakness and leg-load imbalance have been shown to accelerate the development of contralateral OA (72). Supporting evidence from research on anterior cruciate ligament-injured athletes indicates that a 4% reduction in the 5-year probability of clinical OA is associated with each 1% increase in the quadriceps symmetry index, suggesting that achieving symmetric muscle strength may reduce the risk of secondary knee OA (73).

### Symmetrical retraining

Evidence on symmetrical retraining for knee OA is still quite limited. For example, Robadey *et al.* used 3D motion analysis to compare gait patterns in patients with unilateral knee osteoarthritis during a single session of treadmill versus overground running (31). Their findings indicated that running on a treadmill was linked to significantly lower between-limb asymmetry in key gait measures – notably contact time, maximal knee flexion, and vertical speed variance – compared to overground running. Importantly, this reduction in asymmetry appeared to be driven mainly by adjustments in the non-affected limb, which shifted toward the more limited, compensatory movement strategy of the affected side (such as shorter stride length and reduced impact loading), rather than by functional improvement in the affected limb itself. This suggests that the treadmill setting may encourage more symmetrical movement in these patients.

However, as a cross-sectional study, it captured only immediate adaptations rather than long-term changes. The suggested biomechanical mechanisms, while plausible, were not experimentally confirmed. Additionally, the small sample of patients with mild-to-moderate OA limits the generalizability of the findings to broader or more severe cases. Therefore, it is still unclear whether preoperative symmetry-focused retraining provides clear clinical benefits. Future randomized controlled trials are needed to determine if supervised, longer-term retraining can effectively restore normal movement, reduce loading on the opposite limb, and ultimately lower the risk of OA progression or subsequent surgery in the contralateral knee.

Collectively, patients with primary knee OA demonstrate systematic lower-limb asymmetries spanning kinematics, kinetics, and muscle function – such as quadriceps weakness and elevated KAM. Together, these asymmetries form the starting point of a pathomechanical cascade, which is marked by unloading of the more affected limb and a compensatory overload of the contralateral limb, as illustrated in Fig. 1A.

## Asymmetric biomechanics after TKA in patients with knee OA

A growing body of clinical and biomechanical evidence suggests that lasting lower-limb asymmetry after unilateral TKA may be a key mechanism in the progression of OA in the contralateral knee (6, 7, 8). This occurs primarily due to a shift in how weight is distributed, as patients often continue to load the non-operated limb more heavily during daily activities. Gait analysis studies report that the contralateral knee experiences higher dynamic loads relative to the surgical side – a recognized marker of medial compartment loading – and an increased vGRF during stance (5, 74).

At the same time, neuromuscular impairments in the operated limb – particularly insufficient recovery of quadriceps strength and activation – limit its load-bearing capacity. This creates a biomechanical challenge: the surgical limb is unable to share functional loads adequately, leaving the non-operated limb under excessive stress, which may accelerate its degeneration (75, 76).

These factors together form a self-reinforcing cycle. Preferential use of the non-operated limb leads to a sustained mechanical overload, which can accelerate cartilage breakdown through abnormal joint loading. Meanwhile, persistent neuromuscular weakness in the surgical limb further reduces dynamic joint stability, reinforcing the asymmetric movement pattern (70, 77, 78). The following sections evaluate the biomechanical evidence for this mechanism by examining postoperative asymmetries in joint kinematics, kinetics, and muscle function, with a specific focus on the clinical implications for contralateral joint preservation (Fig. 1B).

### Joint kinematics and kinetics after TKA

TKA reliably alleviates pain and improves self-reported function in end-stage knee OA; however, fundamental asymmetries in movement and loading often persist in the long term (79, 80, 81). A characteristic post-TKA profile emerges, featuring reduced flexion of the prosthetic knee, elevated loading on the contralateral limb, knee adduction, and a shortened stride (35, 77, 82).

Objective gait analysis confirms these clinical observations. At six months post-surgery, a significant asymmetry in single-limb support time persists, with less time spent on the operated limb, indicating a continued reliance on the non-operated side during stance (83). This kinetic asymmetry is accompanied by kinematic deviations. Even with bicruciate-retaining implants, the restoration of symmetrical knee motion remains elusive. The operated limb frequently demonstrates excessive flexion and internal rotation during the stance phase compared to the contralateral side (84). Furthermore, inter-limb asymmetry in tibial peak acceleration is more pronounced in TKA patients than in healthy controls (85), and these kinematic patterns exhibit gender-specific variations, with female patients often demonstrating greater asymmetry (86).

The persistence of these abnormal patterns can be attributed to several factors. Preoperatively acquired compensatory strategies for pain avoidance often become ingrained and may persist despite the resolution of pain post-TKA (42, 87). These habitual movement patterns, established to unload the painful knee, can continue to govern motor control even after the pain stimulus has been removed.

From a kinetics perspective, the dynamic asymmetry in joint loading is well documented (72, 88, 89, 90). Significant asymmetries in moments persist in the long term, evidenced by a higher KAM in the non-operated limb six months after surgery (5) and the persistence of moment asymmetries even one year post-TKA, indicating a sustained biomechanical imbalance (91, 92). Studies in elderly populations corroborate this, finding larger heel-strike transients and peak KAMs in the non-operated limb (74).

The evolution of loading asymmetry is dynamic during recovery. In the early postoperative period, patients typically unload the operated limb, which transiently increases loading on the contralateral limb and the ipsilateral hip (83). Christiansen *et al.* outlined this temporal progression: asymmetry peaks around one month, returns to preoperative levels by three months, and often improves further by six months (93). This pattern suggests that the initial imbalance gradually resolves with recovery and adaptation.

In the longer term, however, the picture becomes more complex. Harato *et al.* followed patients for an average of 15 months and found that most (80%) shifted their weight-bearing dominance to the operated limb, while a minority (20%) with extension limitations continued to overload the non-operated side (94). This reversal in the direction of asymmetry highlights the nuanced nature of long-term sensorimotor adaptation. Critically, the core pathological issue is not which side bears more load but the presence of significant asymmetry itself. Any persistent deviation from normal symmetry – whether toward the operated or non-operated limb – can lead to abnormal joint loading. Overloading the non-operated knee directly increases its risk of OA progression, whereas predominant loading on the prosthetic side may indicate unresolved neuromuscular deficits or alter the biomechanics of the implant. Therefore, the primary objective of rehabilitation may lie not in simply shifting load from one side to the other but in addressing underlying neuromuscular deficits and maladaptive movement patterns to promote the restoration of normal, symmetrical biomechanical function.

In summary, the failure to restore biomechanical symmetry after TKA has multiple causes, including retained movement habits, muscle weakness, and the shifting nature of joint loading over time (93, 94, 95, 96). Therefore, the key clinical goal should be restoring functional symmetry – rather than simply redirecting load from one limb to the other – to improve long-term outcomes for both knees.

### Lower-limb muscle strength after TKA

Several studies have reported that patients with knee OA exhibit persistent muscle asymmetry in both lower limbs following TKA. Meier *et al.* observed a 62% reduction in quadriceps muscle strength one month post-TKA compared to preoperative levels, highlighting the significant impact of surgery on muscle function (97). Similarly, Yoshida *et al.* found that three months after unilateral TKA, quadriceps strength on the operated side was markedly weakened, prompting patients to rely more heavily on the non-operated limb during gait. This compensatory mechanism results in greater force exertion on the non-operated leg, further exacerbating muscle asymmetry (70). Over time, reduced mechanical stimulation to the quadriceps on the operated side, due to decreased use in daily activities, may contribute to muscle atrophy and strength deficits (98). Moreover, conventional rehabilitation protocols often do not adequately address persistent neuromuscular deficits, including quadriceps weakness and impaired activation. This insufficient focus on restoring muscle capacity may, therefore, contribute to prolonged strength asymmetry and functional limitations (97).

Despite improvements in self-reported outcomes and quadriceps strength over time, weakness in the quadriceps on the operated side often persists long after TKA (80). Rossi *et al.* reported that, although the asymmetry in knee flexor muscles decreased by less than 10% one year postoperatively compared to preoperative levels, the strength of both flexor and extensor muscles on the affected side ‘still couldn’t catch up’ to the non-affected side, even months or years after surgery. Notably, asymmetry in knee extension and flexion strength between the lower limbs was still evident one year after unilateral TKA (60). These findings underscore the long-term nature of muscle strength deficits following TKA.

Research indicates that restricted joint mobility, not pain, primarily drives asymmetric loading after TKA. Quadriceps strength asymmetry, especially during knee extension, correlates with uneven limb loading (61, 93). Studies show that weight-bearing asymmetry peaks early postoperatively (1 month), is linked to quadriceps strength symmetry rather than pain, and initiates a cycle where limb weakness leads to compensatory loading, reduced use, and further functional decline (61). The asymmetry in lower-limb kinematics and kinetics observed in knee OA patients after TKA is closely linked to quadriceps strength asymmetry (78). Persistent muscle weakness on the operated side can lead to compensatory gait patterns, further perpetuating biomechanical imbalances. Therefore, restoring muscle-level symmetry in knee OA patients after TKA is critical for addressing both kinematic and kinetic asymmetries.

### Symmetrical retraining after TKA

Research on rehabilitation training to improve lower-limb symmetry after TKA is still limited, but early evidence points to its potential for optimizing postoperative movement patterns. Existing studies demonstrate substantial diversity in both intervention timing and methodological approaches.

In the early postoperative phase, Zeni *et al.* introduced symmetry-focused retraining starting 2–4 weeks after surgery and continuing for 6–10 weeks. Their program integrated biofeedback devices – such as the SymSlide and Wii Balance Board – into conventional strength training. At six months, participants demonstrated more symmetrical knee movement in the sagittal plane and loading patterns that more closely resembled those of healthy individuals (99). Supporting this, McClelland’s case report applied an intensive 7-week program between postoperative weeks 3 and 10, combining quadriceps strengthening with biofeedback-guided movement symmetry. This approach led to improved knee flexion range of motion and flexion moments in the surgical limb, while also decreasing excessive load on the nonsurgical side (100).

By contrast, Pötzelsberger *et al.* examined later-stage rehabilitation through a 12-week supervised alpine skiing program in patients more than one year after TKA. Sessions were held 2–3 times weekly, each lasting about 3.5 h, totaling 22–29 ski days over the study period. This ski-based intervention similarly reduced asymmetry between limbs during walking and stair climbing. Importantly, gains were mainly achieved by improving weight-bearing capacity on the operated side, promoting more balanced loading during daily activities (101).

The preliminary evidence from these studies suggests that rehabilitation focused on movement symmetry may aid recovery of the operated limb after TKA (Fig. 1). By addressing muscle imbalances and promoting more even loading between limbs, such training can help minimize compensatory habits and enhance overall movement efficiency. Therefore, rehabilitation programs that target quadriceps strength and restore balance between limbs may improve functional outcomes and lower the risk of further joint problems. This underscores the value of integrating muscle recovery as a central component of postoperative rehabilitation to promote long-term symmetry and function in TKA patients.

However, the limited number of studies in this area represents a significant gap in the literature. Further research is needed to explore and validate additional post-TKA symmetrical retraining methods, with the aim of optimizing rehabilitation protocols and improving long-term outcomes for OA patients. Expanding the scope of such investigations will provide valuable insights into the most effective strategies for restoring lower-limb symmetry and function following TKA.

### Multifactorial pathogenesis of contralateral OA: weighing the biomechanical evidence

While persistent post-TKA biomechanical asymmetries are a key candidate mechanism, it is crucial to recognize that the development and progression of contralateral knee OA results from a combination of multiple factors. Contributing factors typically involve pre-existing early-stage OA in the contralateral knee – a condition that can progress autonomously. This is further amplified by the increased physical activity commonly seen after TKA, owing to successful pain management, which places additional mechanical demand on the joints. Furthermore, systemic issues, such as chronic low-grade inflammation and related metabolic conditions, can also make the contralateral knee more vulnerable to degeneration over time.

Notwithstanding this multifactorial context, biomechanical asymmetry stands as a dominant and modifiable driver. This assertion is supported by the contralateral knee’s disproportionately faster progression compared to other weight-bearing joints in the same patient (8), a pattern best explained by localized overloading. Quantified compensatory mechanisms – a 12–18% vGRF increase and 70% weight-bearing bias (22, 42) – provide a direct pathway for accelerated cartilage wear. Most compellingly, interventions targeting movement symmetry successfully normalize loading and improve function (100, 101), offering a definitive proof of its mechanistic and modifiable role in the disease process.

## Conclusion

Biomechanical factors are central to the initiation and progression of knee OA, with patients often exhibiting significant lower-limb asymmetries, even in bilateral cases. The affected limb typically shows restricted knee flexion and reduced weight-bearing, while the contralateral limb compensates with greater load and mobility. Persistent muscle weakness and joint loading imbalances, particularly in the quadriceps, further exacerbate these asymmetries, potentially driving OA progression in the contralateral knee. Even months to years after TKA, lower-limb asymmetry remains, contributing to a high likelihood of contralateral knee arthroplasty. Rehabilitation programs focusing on restoring bilateral biomechanical symmetry may prevent or delay disease progression by addressing muscle strength imbalances and compensatory mechanisms. Future research should prioritize developing and validating symmetry-focused rehabilitation protocols to improve long-term outcomes for knee OA patients, particularly post-TKA, advancing both biomechanical understanding and clinical management of the disease.

## ICMJE Statement of Interest

The authors declare that they have no known competing financial interests or personal relationships that could have appeared to influence the work reported in this paper.

## Funding Statement

This study was supported by the National Key Research and Development Program of China (2022YFC3601904) and the Key Project of Tianjin Natural Science Foundation (22JCZDJC00340).
